# Peer Review of “Predicting Waist Circumference From a Single Computed Tomography Image Using a Mobile App (Measure It): Development and Evaluation Study”

**DOI:** 10.2196/54045

**Published:** 2023-12-12

**Authors:** William A Barletta

**Affiliations:** 1 Department of Physics, Massachusetts Institute of Technology, Cambridge, MA, United States

**Keywords:** waist circumference, computed tomography, abdominal CT, mobile health, health apps, CT, CT scan, CT image, mobile app, app, application, waist, body, body mass, BMI, morbidity, mortality, clinical, tool, prototype, design, obesity, abdominal, usability, validity, medical

T*his is a peer-review report submitted for the paper “Predicting Waist Circumference From a Single Computed Tomography Image Using a Mobile App (Measure It): Development and Evaluation Study.”*

## Round 1 Review

### General Comments

This paper [1] describes the results of using an author-created app to determine the waist circumference (WC) of patients in both retrospective and anticipatory circumstances. Although the manuscript makes a sound plausibility argument for the use of a smartphone app to determine WC from an existing computed tomography (CT) scan, it offers little rationale for using a pretreatment CT scan in preference to a conventional measurement with a tape measure or equivalent, especially as that measurement modality is taken as the comparison standard.

### Specific Comments

#### Major Comments

 1. The authors admit that their conclusion is based on a very small sample of patients. In recommending further studies, the authors should offer specific guidelines, especially with respect to establishing the precision of each measurement modality. The material speaks only to the accuracy, but the plots in Figures 4 and 5 display some significant outliers.
 2. The manuscript should present quantitative evidence of the degree to which an ellipse is an accurate representation of the body shape at the waist.
 3. The comment that this technique is important to less developed countries is puzzling considering the simplicity and extremely low cost of obtaining tape measure data prior to treatment.
 4. The authors claim that the WC cannot be assessed in patients with intellectual or motor disabilities. Why? That hardly seems like a satisfactory reason to subject the patient to the radiation dose of a CT scan.
 5. Were the statistics presented controlled for variations in BMI and the effect of BMI on the body shape at the waist?

#### Minor Comments

 6. The WC is a characteristic of the patient. It is not a parameter. The text needs careful proofreading.
 7. Unless needed for other clinical reasons, CT scans are not of such limited cost.
 8. In the discussion of statistics, use consistent numbers for significant figures.
 9. In Figures 3 and 4, add the dimensions in the captions.
 10. In the Discussion, why aren’t tape measurements of WC routinely made if this characteristic is so important in treatment planning as the authors claim?
 11. The comment “Also, for a radiologist, conventional CT scan method requires training and can be more or less time consuming” is puzzling in light of the ease of using a tape measure in pretreatment planning.
 12. “Since smartphones are commonly available even in low- and middle-income countries”—CT scanners are not so prevalent. This is a pointless polemic.
 13. In the references, please give PubMed numbers whenever they are available. For websites, give the last date accessed.
 14. The suggestion of using AI in an upgraded app is hardly compelling without a clear explanation of why the ellipse fitting is of questionable validity.

## Abbreviations

CT: computed tomography
WC: waist circumference
